# Association between human T cell leukemia virus 1 (HTLV-1) infection and advanced periodontitis in relation to hematopoietic activity among elderly participants: a cross-sectional study

**DOI:** 10.1186/s12199-019-0796-6

**Published:** 2019-06-10

**Authors:** Yuji Shimizu, Hirotomo Yamanashi, Masayasu Kitamura, Reiko Furugen, Takahiro Iwasaki, Hideki Fukuda, Hideaki Hayashida, Koji Kawasaki, Kairi Kiyoura, Shin-Ya Kawashiri, Toshiyuki Saito, Atsushi Kawakami, Takahiro Maeda

**Affiliations:** 1 Department of Community Medicine, Nagasaki University Graduate School of Biomedical Sciences, Nagasaki-shi, Sakamoto 1-12-4, Nagasaki, 852-8523 Japan; 2 Department of Cardiovascular Disease Prevention, Osaka Center for Cancer and Cardiovascular Diseases Prevention, Osaka, Japan; 3 Department of General Medicine, Nagasaki University Hospital, Nagasaki, Japan; 4 Department of Oral Health, Nagasaki University Graduate School of Biomedical Sciences, Nagasaki, Japan; 5 Department of Community Oral Health, School of Dentistry, Asahi University, Gifu, Japan; 6 Community Medical Network Center, Nagasaki University Hospital, Nagasaki, Japan; 7 Department of Immunology and Rheumatology, Nagasaki University Graduate School of Biomedical Sciences, Nagasaki, Japan; 8 Department of Island and Community Medicine, Nagasaki University Graduate School of Biomedical Sciences, Nagasaki, Japan

**Keywords:** Elderly participants, Hematopoietic activity, HTLV-1, Periodontitis, Reticulocyte

## Abstract

**Background:**

We reported that human T cell leukemia virus 1 (HTLV-1) infection is positively associated with atherosclerosis. Recent evidence has revealed a close association of periodontitis with atherosclerosis, endothelial dysfunction, and disruption of the microcirculation. However, the association between HTLV-1 and advanced periodontitis has not been investigated to date. Since hematopoietic activity is closely linked to endothelial maintenance activity and is known to decline with age, we hypothesized that the state of hematopoietic activity influenced the association between HTLV-1 and advanced periodontitis in elderly participants.

**Methods:**

A cross-sectional study was performed including 822 elderly participants aged 60–99 years who participated in a dental health check-up. Advanced periodontitis was defined as a periodontal pocket ≥ 6.0 mm. Participants were classified as having low or high hematopoietic activity according to the median values of reticulocytes.

**Results:**

HTLV-1 infection was positively related to advanced periodontitis among participants with lower hematopoietic activity (lower reticulocyte count), but not among participants with higher hematopoietic activity (higher reticulocyte count). The adjusted odds ratio (95% confidence interval) considering potential confounding factors was 1.92 (1.05–3.49) for participants with a lower reticulocyte count and 0.69 (0.35–1.36) for participants with a higher reticulocyte count.

**Conclusions:**

Among elderly participants, the association between HTLV-1 infection and advanced periodontitis is influenced by hematopoietic activity. Since hematopoietic activity is associated with endothelial maintenance, these findings provide an efficient tool for clarifying the underlying mechanism of the progression of periodontitis among elderly participants.

## Background

Human T cell leukemia virus 1 (HTLV-1) is an oncogenic retrovirus affecting individuals worldwide, although the southern part of Japan is known as a highly endemic region for HTLV-1 infection [1]. In Japan, the number of HTLV-1 carriers was estimated to be approximately 1.2 million during the late 1980s [2]. The majority of HTLV-1 carriers remain asymptomatic throughout their lives [3–6]. However, we previously identified a positive association of asymptomatic HTLV-1 infection with atherosclerosis [7], which is the result of endothelial dysfunction [8].

In addition, recent studies have revealed that periodontitis is closely associated with atherosclerosis [9], endothelial dysfunction [10, 11], and disruption of microcirculation [12, 13]. Periodontitis is one of the major causes of tooth loss, resulting in a significant decrease in the quality of life of elderly participants [14]; however, the association between HTLV-1 infection and advanced periodontitis among elderly participants remains unknown.

In addition, bone marrow activity (hematopoietic activity) has been recently identified to be associated with endothelial maintenance [15–17]; reticulocyte levels, a marker of hematopoietic activity, were found to be associated with the activity of endothelial maintenance in elderly Japanese individuals [18]. Since aging is also well known to be associated with a reduction in hematopoietic activity as the bone marrow function declines [19–21], hematopoietic activity can be expected to influence the observed association between HTLV-1 infection and advanced periodontitis among elderly participants. Therefore, clarifying the role of reticulocyte levels in mediating the association between HTLV-1 infection and advanced periodontitis could provide an efficient tool for elucidating the mechanism underlying the progression of periodontitis in the elderly.

To evaluate this hypothesis, we conducted a cross-sectional study to examine the influence of hematopoietic activity (assessed by reticulocyte counts) on the association between HTLV-1 and advanced periodontitis among 822 elderly participants that received an annual dental health check-up from 2016 to 2018.

## Methods

### Study population

The study population comprised a total of 1925 participants (702 men and 1223 women) aged 60–99 years from Goto city in the western part of Japan who participated in an annual health check-up with oral assessment in 2016–2018. This annual check-up program is conducted by the local government and directed by the Ministry of Health, Labor and Welfare in Japan. Owing to the shortage of staff to conduct the health check-up in the present survey, the entire city could not be surveyed in 1 year. Therefore, we conducted the survey in different parts of the city over a period of 3 years to ensure that all areas of Goto city were covered. Nevertheless, no individual in the population overlapped among the 3 years of the study. Participants were excluded if there were no carotid intima-media thickness (CIMT) data (*n* = 4) or laboratory data (*n* = 3) available, or if they had less than 10 remaining teeth (*n* = 1078). Since anemia (low levels of hemoglobin, Hb) has been reported to be associated with periodontitis [22, 23] and HTLV-1 infection [24], anemia was considered to be a strong confounding factor for the present analysis; therefore, we excluded participants diagnosed with anemia (Hb < 11 g/dL; *n* = 15). We also excluded participants without available HTLV-1 data (*n* = 3). The remaining participants, comprising 326 men and 496 women, were ultimately included for analysis.

### Data collection and laboratory measurements

A trained interviewer obtained the information on smoking status. Body weight and height were measured while the participant was in bare feet and wearing light clothing using an automatic body composition analyzer (BF-220; Tanita, Tokyo, Japan). Body mass index was calculated as weight (kg)/height (m)^2^.

Blood samples were collected into EDTA-2K tubes and siliconized tubes, and all measurements were conducted according to standard automated laboratory procedures at SRL, Inc. (Tokyo, Japan). The parameters tested included red blood cell (RBC) count, reticulocyte count, Hb level, and serum creatinine level. Reticulocyte count was calculated with the following formula: reticulocytes (× 10^4^ cells/μL) = (reticulocytes, ‰) × RBC (× 10^4^ cells/μL)/1000. The glomerular filtration rate (GFR) was estimated according to an established method with three modifications recently proposed by a working group of the Japanese Chronic Kidney Disease Initiative [25]. According to this adaptation, GFR (mL min/1.73 m^2^) = 194 × [serum creatinine (enzyme method)]^−1.094^ × (age)^−0.287^ × (0.739 for women).

### Measurement of CIMT

CIMT was measured by an experienced vascular technician based on ultrasonography of the left and right common carotid arteries using a LOGIQ Book XP with a 10-MHz transducer (GE Healthcare, Milwaukee, WI, USA). Mean values for the left and right CIMT were calculated with automated digital edge-detection software (Intimascope; MediaCross, Tokyo, Japan) according to a previously described protocol [26]. The values of right and left CIMT, not including plaque measurements, were then calculated, and the mean CIMT value was used for analysis.

### Detection of HTLV-1

A chemiluminescent enzyme immunoassay (CLEIA) kit was used to detect the presence of HTLV-1 (Fujirebio Inc., Tokyo, Japan) according to the manufacturer’s instructions.

### Oral examination

Trained dentists performed a periodontal examination according to a modified method of the Third National Health and Nutrition Examination Survey [27]. Probing pocket depth was measured using a periodontal probe at the mesio-buccal and mid-buccal sites for all present teeth, excluding the third molars. Advanced periodontitis was diagnosed as a probing pocket depth ≥ 6 mm. Prior to the start of the study, all examiners were trained in the same manner and their assessments were calibrated using a chart, periodontal models, and volunteers at Nagasaki University Hospital.

### Statistical analysis

The median value for the reticulocyte count (5.175 × 10^4^ cells/μL for men and 4.926 × 10^4^ cells/μL for women) was set as the cutoff point for classifying the participants into those with low and high hematopoietic activity. The differences in mean values or proportions of reticulocyte levels were analyzed in relation to HTLV-1 status. Significant differences were evaluated using analysis of variance (ANOVA) for continuous variables and chi-squared test for proportion data.

Logistic regression models were used to calculate odds ratios (ORs) and 95% confidence intervals (CIs) to determine the association between HTLV-1 infection and advanced periodontitis. Since aging influences the hematopoietic activity, which in turn influences erythropoietin production, the participants were stratified according to their hematopoietic activity (maintained or reduced hematopoiesis) based on the level of reticulocytes (high or low).

Periodontitis risk factors with a direct influence on the intra-oral environment, such as smoking [28] and number of remaining teeth, were regarded as potential confounding factors in the present analysis. Although no significant correlation between caries (decayed teeth) and periodontitis has been observed in previous studies, both conditions have a common etiology [29, 30]. Therefore, we also included the presence of decayed teeth as a confounding factor in the present analysis. In addition, a low level of Hb can stimulate reticulocyte production and was reported to be associated with more aggressive forms of periodontitis [22], while high levels of Hb are associated with increased arterial stiffness (atherosclerosis) [31]. Since CIMT was reported to be positively associated with periodontitis [9], we included both Hb and CIMT as confounding factors in the model.

In addition, hemoglobin level is known to be associated with renal function [32] as well as increased arterial stiffness, as evaluated by the cardio-ankle vascular index (CAVI) [31]. CIMT is known to be associated with hematopoietic activity [33]. Moreover, hematopoietic activity serves as a determining factor for the association between chronic kidney disease and CIMT [34] and for the association between CAVI and CIMT [35]. Therefore, we also included renal function as a confounding factor in the model, which was evaluated by the GFR. Although endothelial dysfunction is a common initial mechanism leading to atherosclerosis and renal dysfunction [8], the model further adjusted for GFR could present a risk of multicollinearity. Therefore, we established three distinct models to adjust for these confounding factors. The first model was adjusted for only sex and age (model 1); the second model (model 2) included the other potential confounding factors, namely smoking status (never-smoker, former smoker, or current smoker), number of remaining teeth, decayed teeth (yes/no), CIMT (mm), and Hb (g/dL). The last model (model 3) was the one further adjusted for GFR.

All statistical analyses were performed with the SAS system for Windows (version 9.4: SAS Inc., Cary, NC, USA). Values of *p* < 0.05 were regarded as statistically significant.

## Results

### General characteristics of the study population

Among the total 822 participants with a mean ± standard deviation (SD) age of 71.2 ± 6.7 years, 140 (17.0%) showed an HTLV-1-positive status and 181 (22.0%) were diagnosed as having advanced periodontitis; 411 participants had low hematopoietic activity (i.e., a low reticulocyte level).

Table 1 shows the reticulocyte level-specific characteristics of the study population according to the HTLV-1 infection status. No significant associations were observed for participants with lower hematopoietic activity, whereas participants with higher hematopoietic activity and HTLV-1 infection were significantly older than those without HTLV-1 infection.

**Table 1:** Characteristics of study population

	Reticulocyte count levels					
	Low			High		
	HTLV-1 infection		*p*	HTLV-1 infection		*p*
	(−)	(+)		(−)	(+)	
No of subjects	345	66		337	74	
No of advanced periodontitis (%)	72 (20.9)	21 (31.8)	0.052	75 (22.3)	13 (17.6)	0.375
Men, %	40.9	33.3	0.253	39.5	40.5	0.865
Age, year	71.7 ± 7.0	72.8 ± 6.5	0.209	70.2 ± 6.3	72.3 ± 6.5	0.010
Current smoker, %	3.8	7.6	0.167	8.0	4.1	0.237
Former smoker, %	25.8	19.7	0.294	22.0	23.0	0.850
No of remaining teeth	22.3 ± 5.3	23.4 ± 4.9	0.125	22.9 ± 5.2	22.6 ± 4.8	0.574
Individuals with decayed teeth, %	28.1	28.8	0.912	27.9	25.7	0.700
CIMT, mm	0.71 ± 0.13	0.73 ± 0.12	0.321	0.70 ± 0.13	0.72 ± 0.14	0.299
Hemoglobin, g/dL	13.6 ± 1.2	13.4 ± 1.2	0.191	14.1 ± 1.3	13.8 ± 1.1	0.156
Reticulocyte, × 10^4^ cells/μL	3.871 ± 0.744	3.874 ± 0.788	0.978	6.490 ± 1.523	6.747 ± 3.019	0.286
GFR, mL/min/1.73m^2^	70.5 ± 13.1	67.9 ± 14.1	0.135	70.3 ± 15.0	70.7 ± 14.6	0.833

Values: mean ± 1 standard deviation (SD). Advanced periodontitis is defined as periodontal pocket ≥ 6.0 mm. Low reticulocyte levels were < 5.175 × 10^4^ cells/μL for men and < 4.926 × 10^4^ cells/μL for women

*CIMT* carotid intima-media thickness, *GFR* glomerular filtration rate

### Association between advanced periodontitis and HTLV-1

As shown in Table 2, there was no significant association between HTLV-1 infection and advanced periodontitis among the total participants. However, HTLV-1 infection was significantly positively associated with advanced periodontitis for participants with lower hematopoietic activity, but not for those with higher hematopoietic activity (Table 3). In addition, a significant interaction was observed in model 1 and model 2 between HTLV-1 status and hematopoietic activity level (low vs. high) with respect to advanced periodontitis. After further adjustment for GFR (model 3), the interaction showed marginal significance.

**Table 2:** Odds ratios (ORs) and 95% confidence intervals (CIs) for advanced periodontitis in relation to human T cell leukemia virus-1 (HTLV-1) infections

	HTLV-1 infection		*p*
	(−)	(+)	
No of subjects	682	140	
No of case (%)	147 (21.6)	34 (24.3)	
Model 1	1.00	1.15 (0.75, 1.77)	0.523
Model 2	1.00	1.18 (0.76, 1.83)	0.458
Model 3	1.00	1.18 (0.76, 1.83)	0.462

Advanced periodontitis is defined as periodontal pocket ≥ 6.0 mm. Model 1: adjusted only for sex and age. Model 2: adjusted further for smoking status (never, former, current), remained number of teeth, status of decayed teeth (presence, absence), carotid intima-media thickness (CIMT), and hemoglobin. Model 3: adjusted further for glomerular filtration rate (GFR)

**Table 3:** Odds ratios (ORs) and 95% confidence intervals (CIs) for advanced periodontitis in relation to human T cell leukemia virus-1 (HTLV-1) infections by status of reticulocyte count levels

	Reticulocyte count levels						Interaction
	Low			High			
	HTLV-1 infection		*p*	HTLV-1 infection		*p*	*p*
	(−)	(+)		(−)	(+)		
No of subjects	345	66		337	74		
No of case (%)	72 (20.9)	21 (31.8)		75 (22.3)	13 (17.6)		
Model 1	1.00	1.85 (1.03, 3.32)	0.040	1.00	0.67 (0.34, 1.30)	0.238	0.036
Model 2	1.00	1.92 (1.05, 3.49)	0.034	1.00	0.69 (0.35, 1.36)	0.283	0.039
Model 3	1.00	1.89 (1.04, 3.45)	0.038	1.00	0.70 (0.35, 1.37)	0.295	0.050

Advanced periodontitis is defined as periodontal pocket ≥ 6.0 mm. Low reticulocyte levels were <5.175×10^4^ cells/μL for men and <4.926×10^4^ cells/μL for women. Model 1: adjusted only for sex and age. Model 2: adjusted further for smoking status (never, former, current), remained number of teeth, status of decayed teeth (presence, absence), carotid intima-media thickness (CIMT), and hemoglobin. Model 3: adjusted further for glomerular filtration rate (GFR)

Sensitivity analysis according to the quartile of reticulocyte levels demonstrated associations between HTLV-1 infection and advanced periodontitis, similar to the main results.

## Discussion

The major finding of the present study is that HTLV-1 infection is positively associated with advanced periodontitis only among elderly participants with lower hematopoietic activity (a lower reticulocyte count).

A previous case-controlled cross-sectional study including 42 periodontally healthy individuals, 64 HTLV-1-seronegative individuals presenting with chronic periodontitis, and 36 individuals with chronic periodontitis with HTLV-1-seropositive HTLV-1-associated myelopathy (HAM)/symptomatic tropical spastic paraparesis (TSP) revealed that HTLV-1 may play a critical role in the pathogenesis of periodontal disease through deregulation of the local cytokine network [36].

In the present study, we found further evidence that HTLV-1 infection is positively associated with advanced periodontitis among participants with low reticulocyte levels, even though most of the participants were asymptomatic.

Periodontitis has been shown to be closely associated with atherosclerosis [9], endothelial dysfunction [10, 11], and disruption of the microcirculation [12, 13], and low levels of erythrocytes and Hb are positively associated with periodontitis [22, 23]. In addition, reticulocyte levels are reported to be inversely associated with CIMT in elderly Japanese individuals [18]. Therefore, maintaining higher levels of hematopoietic activity (i.e., a high reticulocyte level) should protect against the progression of periodontitis in elderly participants. However, we found a similar prevalence of advanced periodontitis in participants with low (22.6%) and high (21.4%) hematopoietic activity, as assessed by the reticulocyte count.

Aging is known to be associated with a decline of bone marrow activity (reduction in hematopoietic activity) [19–21] and also contributes to endothelial dysfunction via increasing oxidative stress [37, 38]. Since the hematopoietic activity in elderly participants is determined by both of these age-related factors, higher hematopoietic activity can help to maintain endothelial function even if the magnitude of endothelial damage is severe [17, 18]. In other words, elderly participants with lower hematopoietic activity would have more difficulty in maintaining endothelial function, regardless of the severity of age-related endothelial dysfunction. We previously reported an ambivalent association of reticulocytes in hypertension and atherosclerosis (endothelial dysfunction), in which hypertension and atherosclerosis showed a significantly positive association; however, hypertension showed a significantly positive association with reticulocytes while atherosclerosis showed a significantly inverse association with reticulocytes [18]. The present study partly supports the abovementioned mechanism by indicating that the reticulocyte level serves as an indicator of not only endothelial damage but also endothelial repair activity. Furthermore, this study suggests that individuals with high levels of reticulocytes should have a higher capacity of endothelial maintenance compared to those with low levels of reticulocytes.

In addition, HTLV-1 infection is reported to be positively associated with atherosclerosis [7]. Therefore, compared to participants without HTLV-1 infection, HTLV-1-infected participants might have a greater degree of endothelial dysfunction, which can increase the risk of periodontitis [9–13]. This relationship between hematopoietic activity and endothelial function explains the significantly positive association between HTLV-1 infection and advanced periodontitis only in participants with lower hematopoietic activity (lower endothelial repair activity).

Furthermore, a significant interaction was observed between HTLV-1 status and hematopoietic activity level (low vs. high) with respect to advanced periodontitis. These results also support the abovementioned mechanism by indicating that hematopoietic activity levels could act as a determinant of the association between HTLV-1 and advanced periodontitis.

Some potential limitations of this study warrant consideration. Although the age-related endothelial dysfunction should have an influence on the association between HTLV-1 infection and advanced periodontitis, no data on endothelial function were available to directly assess this relationship. Thus, further analyses that include endothelial function-related data such as flow-mediated dilation [39] will be necessary.

Owing to the lack of knowledge regarding the determination of the exact cutoff point, the median values of reticulocytes were used for the present analysis. Further investigation is necessary to clarify whether the balance between endothelial damage and endothelial repair that contributes to endothelial maintenance can help to determine the exact cutoff point for the present associations. In addition, because this was a cross-sectional study, causal relationships could not be established. Nevertheless, the association identified provides new insights into the potential mechanisms mediating the relationship between HTLV-1 and periodontitis, highlighting the importance of maintaining hematopoietic activity for the overall health of elderly participants.

## Conclusions

HTLV-1 infection is positively associated with advanced periodontitis among elderly participants with lower hematopoietic activity (a lower reticulocyte count) but not among elderly participants with higher hematopoietic activity (a higher reticulocyte count). Since elderly participants with higher hematopoietic activity should have higher tissue-repairing activity [40] with a protective effect against periodontitis progression [23], these findings can help to clarify the underlying mechanism contributing to the progression of periodontitis among elderly participants.

## Data Availability

The datasets generated during and/or analyzed during the current study are not publicly available due to ethical consideration but are available from the corresponding author on reasonable request.

## Abbreviations

ANOVA: Analysis of variance
CIMT: Carotid intima-media thickness
CIs: Confidence intervals
CLEIA: Chemiluminescent enzyme immunoassay
HAM: HTLV-1-associated myelopathy
Hb: Hemoglobin
HTLV-1: Human T cell leukemia virus 1
ORs: Odds ratios
RBC: Red blood cell
SD: Standard deviation
TSP: Tropical spastic paraparesis
